# Pan-cancer analysis identified OAS1 as a potential prognostic biomarker for multiple tumor types

**DOI:** 10.3389/fonc.2023.1207081

**Published:** 2023-09-06

**Authors:** Shan Jiang, Xinzhou Deng, Ming Luo, Le Zhou, Jingjing Chai, Chao Tian, Yutao Yan, Zhiguo Luo

**Affiliations:** ^1^ Department of Clinical Oncology, Taihe Hospital, Hubei University of Medicine, Shiyan, Hubei, China; ^2^ Hubei Key Laboratory of Stem Cell Research, Taihe Hospital, Hubei University of Medicine, Shiyan, Hubei, China

**Keywords:** OAS1, biomarker, prognosis, pan-cancer, tumor immune response

## Abstract

**Background:**

2’,5’-oligoadenylate synthetase 1 (OAS1), has been reported as a tumor driver gene in breast carcinoma and pancreatic carcinoma. However, the role of OAS1 in most tumors has not been reported.

**Methods:**

The original data of 35 tumor types were down load from the TCGA (The Cancer Genome Atlas) database and Human Protein Atlas (HPA) database. TIMER2, Kmplot, UALCAN, and TISIDB tools were used to investigate the expression and function of OAS1, and the role of OAS1 in prognosis, diagnostic value, and immune characteristics of pan-cancer. LUAD and PRAD cell lines, A549, H1975, PC-3 and C4-2 were utilized to perform cell function tests.

**Results:**

OAS1 expression was up-regulated in 12 tumor types and down-regulated in 2 tumor types. High OAS1 expression was correlated with poor prognosis in 6 tumor types, while high OAS1 expression was correlated with good prognosis in 2 tumor types. OAS1 was correlated with molecular subtypes in 8 tumor types and immune subtypes in 12 tumor types. OAS1 was positively associated with the expression of numerous immune checkpoint genes and tumor mutational burden (TMB). OAS1 had potential diagnostic value in 15 tumor types. Silence of OAS1 significantly inhibited the cell proliferation ability, and promoted G2/M cell cycle arrest of LUAD and PRAD cells. Meanwhile, silence of OAS1 enhanced cisplatin-induced apoptosis of LUAD and PRAD cells, but weakened cell migration.

**Conclusion:**

This pan-cancer study suggests that OAS1can be used as a molecular biomarker for prognosis in pan-cancer and may play an important role in tumor immune response.

## Introduction

For a long time, cancer has been a serious threat to human life and health (1). Due to the lack of effective and specific diagnostic markers and the low early diagnosis rate, most of them are already in the advanced stage at the time of diagnosis, leading to their poor treatment effect, poor prognosis and high mortality rate of patients (2). Tumor treatments contain surgery, chemoradiotherapy, targeted therapy and immunotherapy (3). Despite rapid progress in cancer treatment during the last two decades, the therapeutic effect and prognosis of most tumor patients with advanced-stage are still not ideal. Therefore, there is an urgent need to explore the new targets for tumor diagnosis and therapy.

OAS1 (2’,5’-oligoadenylate synthetase 1) is one of the main members of the OAS family and an important antiviral enzyme produced by interferon induction (4, 5). The OAS1 gene, which is located on chromosome 12 and plays a role in the human innate immune response against viral infection (6). IFN binding to specific antibodies cause the activation of OAS1, activated OAS1 promotes the synthesis of 2-5A and activation of RNA enzyme L, which is involved in cell apoptosis, differentiation and protein synthesis (7, 8). OAS1 may participate in the development of autoimmune diseases (9), such as type 1 diabetes (10) and systemic lupus erythematosus (11). In 2022, several studies found that OAS1 was an effector gene affecting the severity of COVID-19 in Europe and Africa (12, 13). In recent years, studies showed that OAS1 also plays some roles in tumors. It has been shown that OAS1, as a key regulated gene in breast cancer, and upregulated in breast cancer patients (14). OAS1was upregulated in pancreatic cancer and was correlated with poor prognosis in patients (15). Moreover, some reports have showed that OAS1 is involved in the regulation of tumor cell apoptosis. To date, the expression level and role of OAS1 in most tumors are still unclear, and a comprehensive analysis of the functional and clinical significance of OAS1 at the pan-cancer level has not yet been performed.

This study systematically investigated the correlation of OAS1 expression level with the prevalence and prognostic value at the pan-cancer level. We used multiple databases, including TCGA, UALCAN, GEPIA, TIMER2, and TISIDB, to comprehensively evaluate the effect of OAS1 in prognosis and immune response in pan-cancer. OAS1 was upregulated in 12 tumor types and downregulated in 2 tumor types. OAS1 was correlated with overall survival (OS) and disease-free survival (DFS) in multiple tumors. OAS1 expression levels are highly correlated with multiple immune checkpoint genes and TMB. Our cellular experiments also implicated that OAS1 plays a supportive role in tumor progression. This study suggests that OAS1 is a promising biomarker for the diagnosis and prognosis in pan-cancer, and can serve as a potential molecular target for multiple cancers.

## Materials and methods

### Gene expression analysis

The original data were downloaded from the TCGA database (https://cancergenome.nih.gov). TIMER2 (http:///timer.cistrome.org/) (16) was utilized to detect OAS1 expression. Based on the TCGA database, we downloaded the transcriptome data of the pan-cancer patients and the corresponding clinical data, and collated the clinical data using the R software. Then, bioinformatics analysis was used to explore the expression level of OAS1 in cancer tissues and normal tissues. UALCAN database (http://ualcan.path.uab.edu//analysisprot.html) was utilized to assess protein expression levels of OAS1 in pan-cancers. The GEPIA database (http://GEPIAcancer-pku.cn) (17) was utilized to detect the expression level of OAS1 in different tumor stages.

### Immunohistochemical analysispan-cancers

The Human Protein Atlas (HPA) (http:///www.proteinatlas.org/) database was utilized to evaluate the protein expression levels of OAS1 in tumor and adjacent tissues. Immunohistochemical images of six tumor tissues and corresponding normal tissues were obtained from HPA database, including BRCA (breast cancer), COAD (colon cancer), THCA (thyroid cancer), LUAD (lung adenocarcinoma), HNSC (head and neck squamous cell carcinoma) and UCEC (endometrial cancer).

### OAS1 expression in molecular subtypes or immune subtypes of pan-cancers

We detected the relationships between OAS1 expression and molecular subtypes and immune subtypes from the TISIDB database (18). TISIDB is a public, open and multiduty database to analyze the interactions between tumors and the immune system.

### Survival analysispan-cancers

In this study, we used the kmplot database (http://kmplot.Thecom/analysis/) (19) and GEPIA databases to perform the OS and DFS analysis. These two databases are public databases that are often used to analyze gene expression levels as well as survival time of patients (20).

### The receiver operating characteristic analysispan-cancers

The ROC curve was utilized to detect the diagnostic value of OAS in various tumors using R software (R-4.0.9). The closer the AUC value is to 1, the better the reference value for diagnosis.

### KEGG and GSEA analysespan-cancers

In this study, to analyze the biological processes and signaling pathways that regulated by OAS1 in tumors, the Linked Omics database (http://www.linkedomics.org) (21) was utilized to perform KEGG analysis and GSEA analysis using R software (R-4.0.9). Pearson’s correlation was used to analyze the relation between OAS1 and co-expression genes.

### Correlation analysis of OAS1 expression and immune checkpoint genes

The data of cancer and normal tissues of pan-cancer were downloaded from TCGA. R software v4.0.9 was utilized to generate a heat map of Spearman correlations to explore the correlation between multiple immune checkpoint genes and OAS1 expression in pan-cancers.

### Tumor mutational burden analysis

GDC (https://portal.gdc.camcer.gov/) database was utilized to obtain the Simple Nucleotide Variation data, and calculated the TMB of each cancer types using R software (R-4.0.9).

### Cell culture and transfection

Lung cancer cell lines (H1975 and A549) and prostate cancer cell lines (PC-3 and C4-2) were purchased from ATCC. Cell lines were cultured in DMEM (Gibco, Carlsbad, CA, USA) containing 10% fetal bovine serum (BI) and maintained at 37°C with 5% CO_2_. Allowed the cells to grow to ~70% confluency, and 1×10^5^cells/well were inoculated to 6-well plates (Corning, NY, USA) one day before transfection (22). The riboFECTTMCP Transfection Kit (ribobio, Guangzhou, China) was utilized to transfect siRNA into cells. The siRNA sequences of OAS1 used were: negative siRNA negative control (NC) (5’-TTCTCCGAGCGTCACGT-3’), siRNA-OAS1 #1 (5’-CCGCAUGCAAAUCAA CCAUTT-3’), siRNA-OAS1 #2 (5’-GCUUCCGAGGUAGCUCCUATT-3’), and siRNA-OAS1 #3 (5’-GGUGGAGACCCAAAGGGUUTT -3’).

### Cell proliferation assay

96-well plates were used to inoculate cancer cells at 1500 cells/well after cells transfected with OAS1-siRNA, and cells were cultured for the indicated days. Each well of 96-well plates were added 10μl Cell Counting Kit 8 (CCK-8, Dojindo) and incubated for 1.5 hours (23). Microplate reader (Bio-Rad, Gaithersburg, MD, USA) was used to measure the absorbance at 450 nm.

### Western blot assay

The cells were transfected with OAS1 specific siRNA for 72 h and then lysed with RIPA (medchemexpress), then protein lysates were boiled at 100˚C with 5×loading buffer for 6 min. The cell lysates were separated using 10% SDS-PAGE and transfer to the PVDF membranes (Millipore, USA) (24). The PVDF membranes were blocked with 5% defatted milk for 2 h. The anti-OAS1 (Santa Cruz, CA, sc-374656, 1:2000 dilution), anti-GAPDH (BOSTER, China, A00227-1, 1:4000 dilution) were used as primary antibody. HRP-conjugated goat anti-mouse (BOSTER, China, BA1051, 1:5000 dilution) was used as secondary antibody. ImageJ software was used for stripe analysis.

### Cell cycle analysis

Cancer cells were digested and resuspension, and washed twice with PBS after cells transfected with siRNA for 72 h. Cells were fixed with 70% precooled ethanol at 4°C for 1 day. The next day, the cells were washed three times with PBS, then stained with Cell Cycle Kit (Beyotime, China, C1052) (25). The cells were then analyzed by a flow cytometry (Beckman Coulter Quanta SC System).

### Cell apoptosis assay

Cells were seeded in wells of 6-well plates with 15×10^4^cells/well and incubated in DMEM containing 10% FBS. The OAS1-siRNAs were used to impair OAS1 expression for 48 h, then cells were treated with 10 μM cisplatin for 24h. Finally, cells were digested and re-suspension with 1×bingding buffer, then, cells were treated with Annexin V-FITC (10 μl) and PI (5μl) (Absin, China) (26).

### Transwell assay

Cancer cells were transfected with OAS1 specific siRNA for 48 h. Then cells were digested and suspension in DMEM with 0.1% BSA, 4×10^4^cells/well was inoculated into the upper chambers (Costar, MA, USA), the lower chambers were added 500μl DMEM plus 10% FBS. Cells were allowed to migrate for 12 h in incubator. Use a cotton swab to wipe away the cells in the upper chamber before staining. The migrated cells were fixed with methanol and 0.1% crystal violet was utilized to stain cells (27), the migrated cells were photographed and counted using a microscope (Olympus).

### Statistical analysis

Student’s t-test was used to evaluate alterations of OAS1 expression levels in tumor tissue and normal tissue. Kruskal test was used for multiple sets of samples. Spearman or Pearson test was used to analyze the correlation between the two variables. Kaplan-Meier curves were used to perform survival analysis, Cox regression was used to assess the HR and p value. The R language software (R-4.0.9) was utilized to perform statistical analysis in this study. P<0.05 was considered statistically significant. pan-cancers

## Results

### Expression analysis of OAS1 in pan-cancer

**Figure 1:**
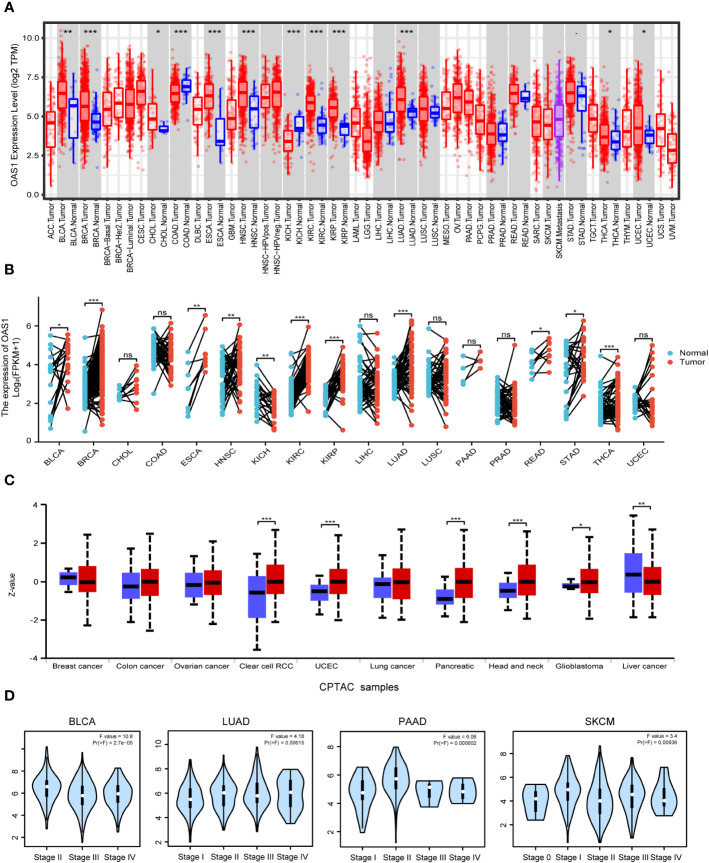
Expression level of OAS1 in human tumors. **(A)** Transcription level of OAS1 in unmatched tumor tissues vs adjacent normal tissues (if available) as visualized using TIMER2. **(B)** Transcription level of OAS1 in matched tumor tissues and adjacent normal tissues. **(C)** The total protein level of OAS1 in normal tissue and cancer tissues using UALCAN database. **(D)** Transcription level of OAS1 in pathological stages of BLCA, LUAD, PAAD and SKCM using TCGA data. *p < 0.05, **p < 0.01, ***p < 0.001. ns: no significance.

We used the TIMER 2 database to analyze the expression level of OAS1 in pan-cancer. We found an inconsistent expression of OAS1 expression in pan-cancer. In BLCA (Bladder Urothelial carcinoma), BRCA, CHOL (Cholangiocarcinoma), ESCA (Esophageal carcinoma), HNSC, KIRC (Kidney Renal Clear Cell carcinoma), KIRP (Kidney Renal Papillary Cell carcinoma), LUAD, THCA and UCEC, OAS1 expression was significantly higher in tumor tissues than that in adjacent normal tissues, whereas OAS1 expression was significantly lower in COAD and KICH (Kidney Chromophobe) than that in adjacent normal tissues (**Figure 1A**). Meanwhile, in order to comprehensively analyze the expression of OAS1 in pan-cancer, the expression level of OAS1 in tumor tissues and matched normal tissues were also examined using TCGA database. ESCA, BLCA, BRCA, HNSC, KIRC, KIRP, LUAD, READ (Rectum adenocarcinoma), STAD (Stomach adenocarcinoma) and THCA show high expression of OAS1 in tumor tissues than that in matched normal tissues, whereas OAS1 mRNA level in KICH was significantly lower than that in the matched normal tissues (**Figure 1B**).

**Figure 2:**
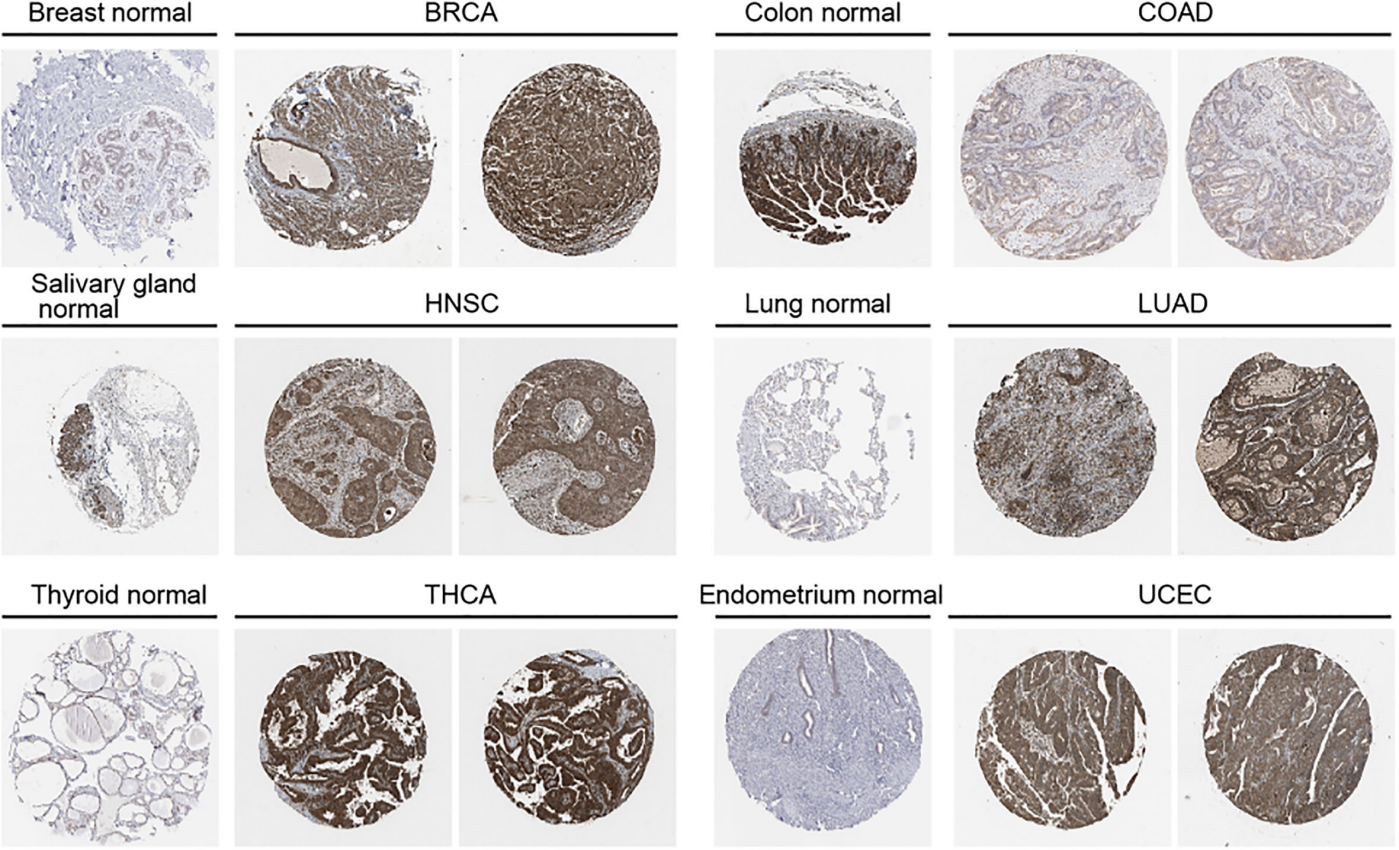
Protein expression level of OAS1 in normal and tumor tissues using immunohistochemistry images from HPA database.

We further used the UALCAN database to explore OAS1 protein expression levels in tumor tissue and the corresponding normal tissue. OAS1 protein levels were up-regulated in breast cancer, COAD, ovarian cancer, renal clear cell carcinoma, endometrial cancer, lung cancer, pancreatic cancer, HNSC and glioblastoma, but down-regulated in liver cancer (**Figure 1C**). We also analyzed immunohistochemical results using HPA database. In BRCA, HNSC, HUAD, THCA, and UCEC, tumor tissues show higher expression level of OAS1 than that in normal tissues, but OAS1 was lower in tumor tissues than that in normal tissues in COAD (**Figure 2**).

We also examined the relationship between OAS1 expression and tumor pathological stage using the GEPIA tool. Results showed that OAS1 expression was associated with several tumor pathologic stage, including BLCA, LUAD, PAAD (Pancreatic adenocarcinoma) and SKCM (Skin Cutaneous Melanoma) (**Figure 1D**).

### Correlations between OAS1 and molecular subtypes or immune subtypes of pan cancers

**Figure 3:**
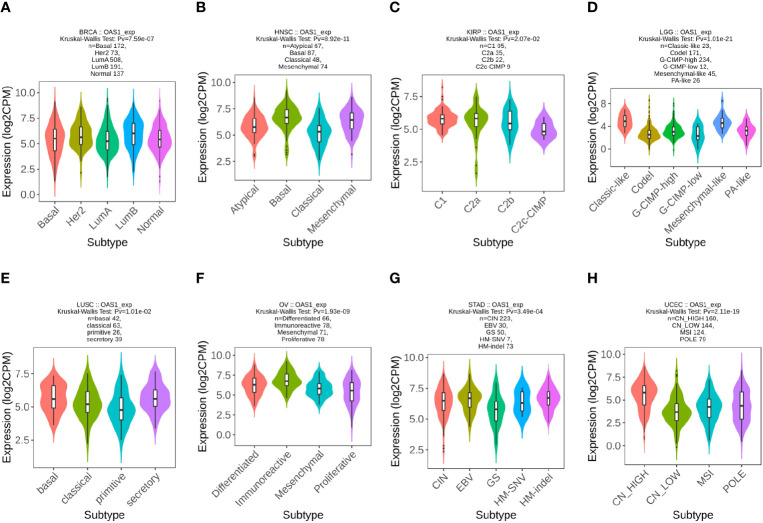
The expression level of OAS1 in different molecular subtypes in pan-cancers using TISIDB database. **(A)** BRCA; **(B)** HNSC; **(C)** KIRP; **(D)** LGG; **(E)** LUSC; **(F)** OV; **(G)** STAD;**(H)** UCEC.

We further examined the expression levels of OAS1 in tumor patients with different molecular subtypes in pan-cancers. We observed that the expression levels of OAS1 were different in diverse molecular subtypes of eight cancer types. In BRCA, OAS1 express was higher in the molecular subtype of LumB than others (**Figure 3A**). In HNSC, OAS1 showed highest expression level in the molecular subtype of Basal (**Figure 3B**). In KIRP, OAS1 showed higher expression level in the molecular subtype of C1 and C2a than C2b and C2c-CIMP (**Figure 3C**). In LGG, OAS1 express was highest in the molecular subtype of Classic-like than others (**Figure 3D**). In LUSC, OAS1 showed higher expression level in the molecular subtype of basal and secretory than classic and primitive (**Figure 3E**). In OV, OAS1 express was highest in the molecular subtype of Classic-like than others (**Figure 3F**). In STAD, OAS1 showed higher expression level in the molecular subtype of EBV and HM-indel than CIN, GS and HM-SNV (**Figure 3G**). In UCEC, OAS1 express was highest in the molecular subtype of CN-HIGH than others (**Figure 3H**). These data indicate that the expression of OAS1 was associated with molecular typing of tumors.

**Figure 4:**
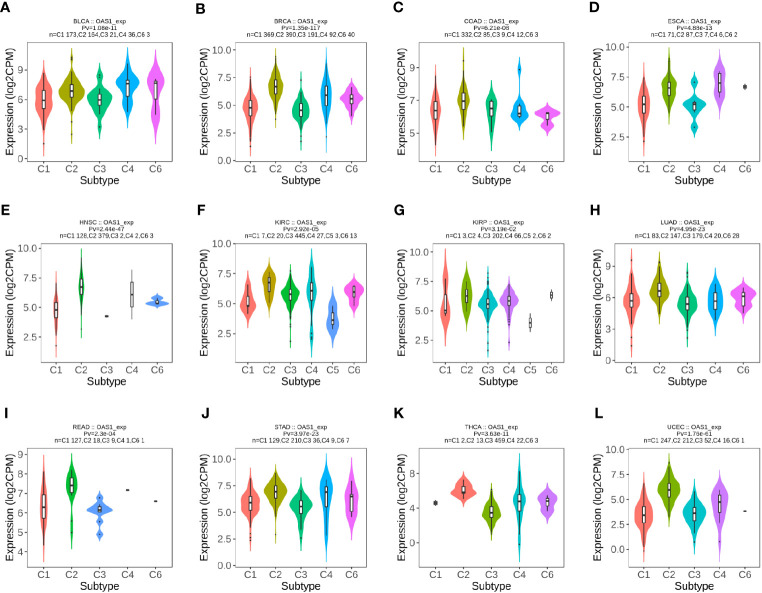
The expression level of OAS1 in different immune subtypes in pan-cancers using TISIDB database. **(A)** BLCA; **(B)** BRCA; **(C)** COAD; **(D)** ESCA; **(E)** HNSC; **(F)** KIRC; **(G)** KIRP; **(H)** LUAD; **(I)** READ; **(J)** STAD; **(K)** THCA; **(L)** UCEC.

In order to explore the correlation between OAS1 and tumor immune subtypes, the TISIDB database was used to explore the expression of OAS1 in different immune subtypes of pan-cancers. We found different expression of OAS1 in 6 different immune subtypes of tumors (C1: Wound Healing, C2: IFN-γ Dominant, C3: Inflammatory, C4: Lymphocyte Depletion, C5: Immunologically Quiet, C6: TGF-β Dominant). OAS1 was found highest in BLCA (**Figure 4A**) and KIRP (**Figure 4G**) with C6 signature. OAS1 was highest in BRCA (**Figure 4B**), COAD (**Figure 4C**), HNSC (**Figure 4E**), KIRC (**Figure 4F**), LUAD (**Figure 4H**), READ (**Figure 4I**), STAD (**Figure 4J**), THCA (**Figure 4K**), and UCEC (**Figure 4L**) with the interferon gamma (IFN-γ) dominant (C2) signature. OAS1 was highest in ESCA (**Figure 4D**) with the lymphocyte depleted (C4) signature. Reports show that C2 signature is correlated with less favorable outcomes, C4 and C6 are correlated with the least favorable outcome. These data suggest that OAS1 may be correlated with tumor immune status and as an oncogene in multiple tumors.

### The correlation between OAS1 expression and prognosis in pan-cancer

**Figure 5:**
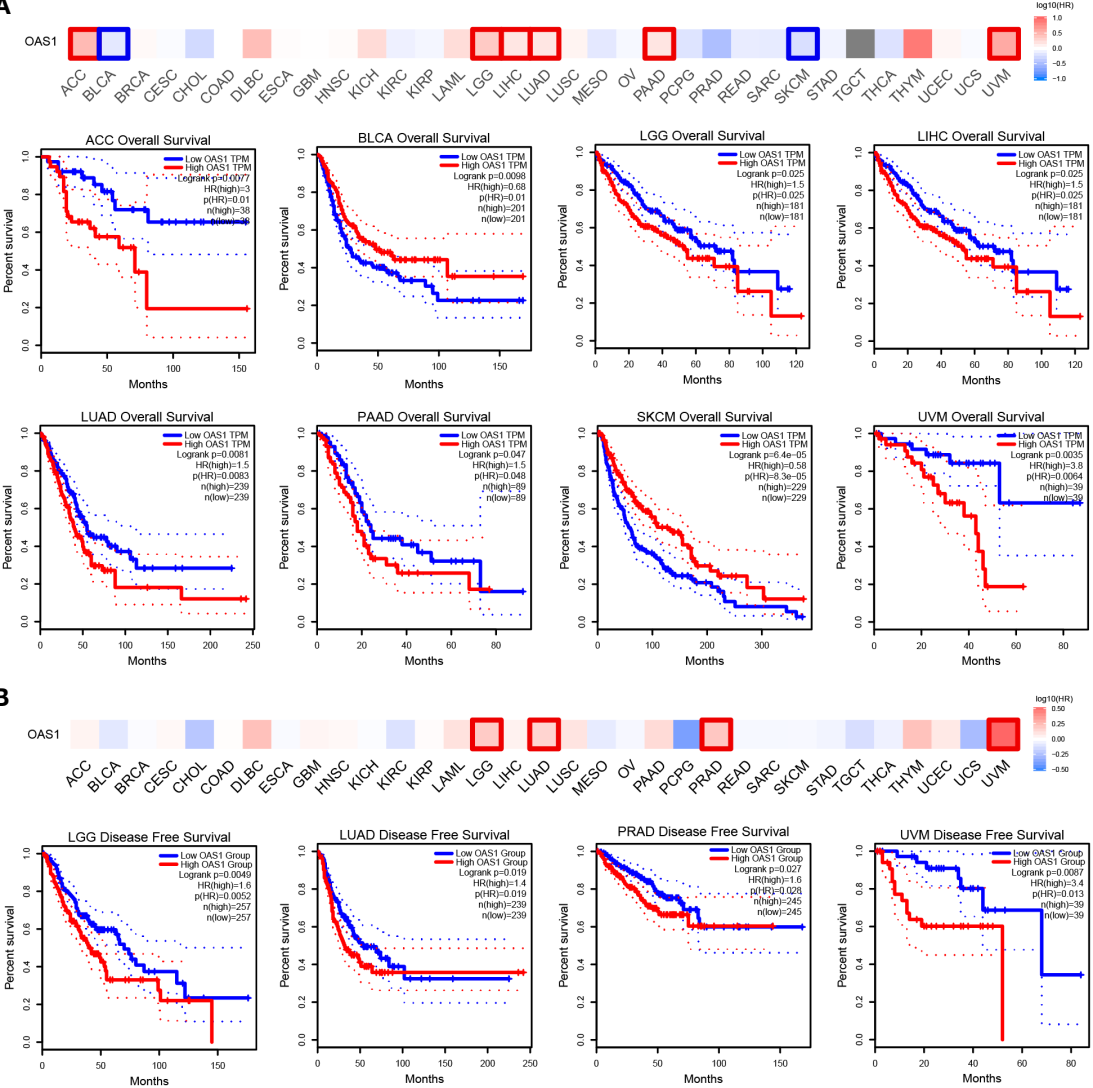
Kaplan–Meier survival curve explore correlation between OAS1 expression and survival rates in patients with different TCGA tumor types using GEPIA. **(A)** OS, **(B)** DFS. Statistically significant survival curves are presented.

Next, we focused on the correlation between OAS1 expression and patient prognosis. According to the expression level of OAS1, tumor patients were divided into high and low OAS1 expression groups. Patients with high expression of OAS1 showed poor OS (overall survival), including ACC (Adrenocortical carcinoma) (P=0.0077), LGG (Brain lower grade glioma) (P=0.025), LIHC (P=0.025), LUAD (P=0.0081), PAAD (P=0.047), and UVM (Uveal Melanoma) (P=0.0035). Whereas patients with high expression of OAS1 showed longer OS, including BLCA (P=0.0098) and SKCM (P=0.000064) (**Figure 5A**). In addition, high OAS1 expression was associated with poor DFS (disease-free survival), including LGG (P=0.0049), LUAD (P=0.019), PRAD (Prostate adenocarcinoma) (P=0.027), and UVM (P=0.0087) (**Figure 5B**).

### The diagnostic value of OAS1 in pan-cancer

**Figure 6:**
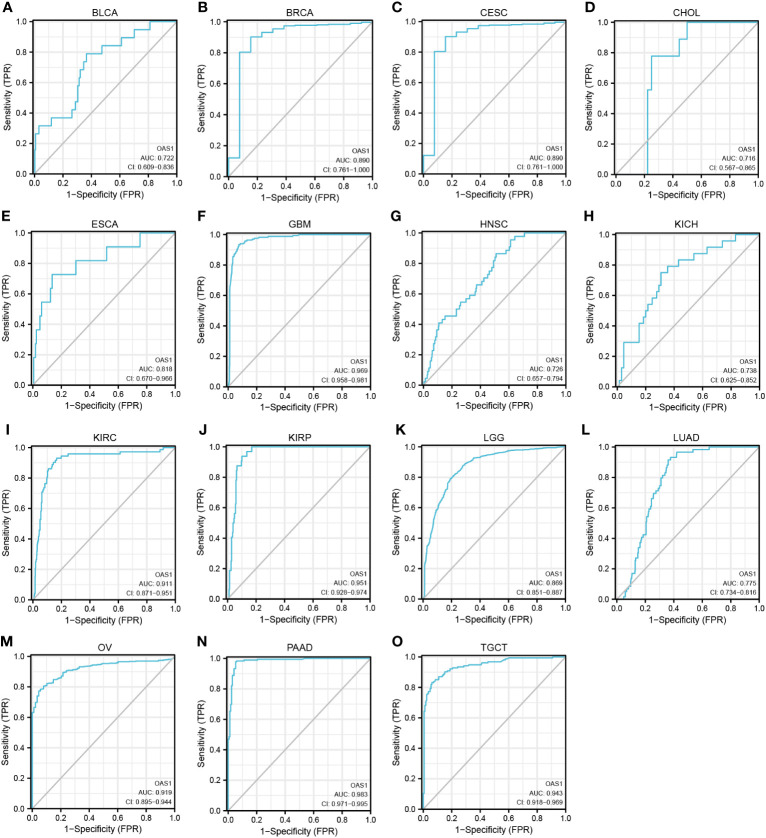
ROC curve for OAS1 expression in pan-cancer. **(A)** BLCA; **(B)** BRCA; **(C)** CESC; **(D)** CHOL; **(E)** ESCA; **(F)** GBM; **(G)** HNSC; **(H)** KICH; **(I)** KIRC; **(J)** KIRP; **(K)** LGG; **(L)** LUAD; **(M)** OV; **(N)** PAAD; **(O)** TGCT.

ROC curves were used to evaluate the diagnostic value of OAS1 in pan-cancer. We observed that OAS1 had a certain reference value in the diagnosis of 15 cancer types (AUC> 0.7), including BLCA (AUC = 0.722) (**Figure 6A**), BRCA (AUC = 0.890) (**Figure 6B**), CESC (AUC = 0.890) (**Figure 6C**), CHOL (AUC = 0.716) (**Figure 6D**), ESCA (**Figure 6E**), GBM (AUC = 0.969) (**Figure 6F**), HNSC (AUC = 0.726) (**Figure 6G**), KICH (AUC = 0.738) (**Figure 6H**), KIRC (AUC = 0. 911) (**Figure 6I**), KIRP (AUC = 0.951) (**Figure 6J**), LGG (AUC = 0.869) (**Figure 6K**), LUAD (AUC = 0.775) (**Figure 6L**), OV (AUC = 0.919) (**Figure 6M**), PAAD (AUC = 0.983) (**Figure 6N**), TGT (AUC = 0.943) (**Figure 6O**). Among them, OAS1 predicted GBM, KIRC, KIRP, OV, and PAAD with a high accuracy (AUC> 0.9).

### Functional enrichment analysis of the OAS1 in pan-cancer

**Figure 7:**
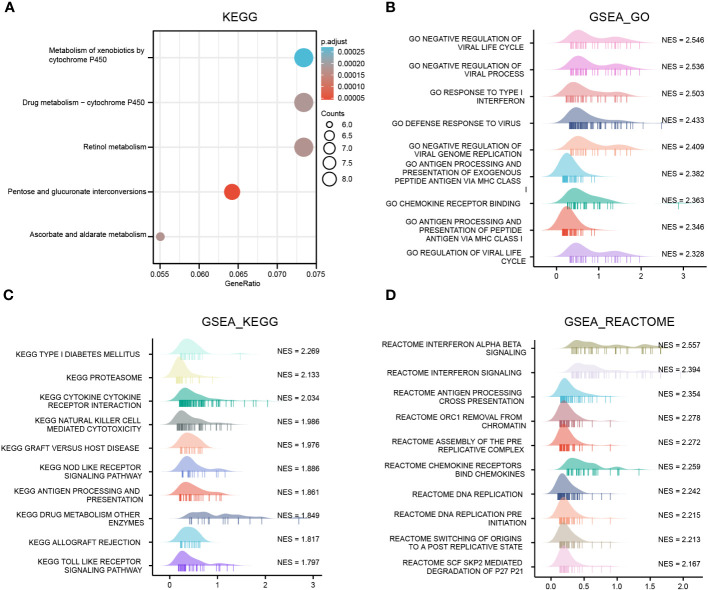
OAS1 functional clustering using KEGG and GSEA annotation in pan-cancers. **(A)** KEGG annotation analysis, **(B)** GSEA_GO annotation analysis, **(C)** GSEA_ KEGG, **(D)** GSEA_REACTOME annotation analysis.

To investigate the biological processes and molecular mechanisms of OAS1 regulation in pan-cancer, we identified a gene-sets including top200 genes that positively co-expressed with OAS1 using GEPIA database, which were used to perform GSEA and KEGG annotation analyses. The top 5 pathways of KEGG analysis included metabolism of xenobiotics by cytochrome P450, retinol metabolism, pentose and glucuronate interconversions, and ascorbate and aldarate metabolism (**Figure 7A**). GSEA_GO annotation was used to assess the OAS1-related signaling pathways that were differentially activated in pan-cancer. Data showed that OAS1 affects multiple gene ontology (GO) pathways, including negative regulation of the viral life cycle and process, response to type I interferon, defense response to virus, antigen processing and presentation of exogenous peptides antigen *via* MHC class, and chemokine receptor binding (**Figure 7B**).

GSEA_KEGG analysis suggested that OAS1 was involved in type I diabetes, proteasome, cytokine receptor interaction, NK cell-mediated cytotoxicity, graft-versus-host disease, NOD like receptor signaling pathways, processing and presentation of antigen (**Figure 7C**). GSEA_REATOME analysis indicated that several immune functional terms were enriched in the gene sets co-expressed with OAS1, including interferon signaling, antigen processing cross presentation, chemokine receptors binding chemokines (**Figure 7D**). These results suggest that OAS1 was involved in antiviral response and the immune response.

### Correlation analysis of OAS1 expression and immune checkpoint genes and TMB

**Figure 8:**
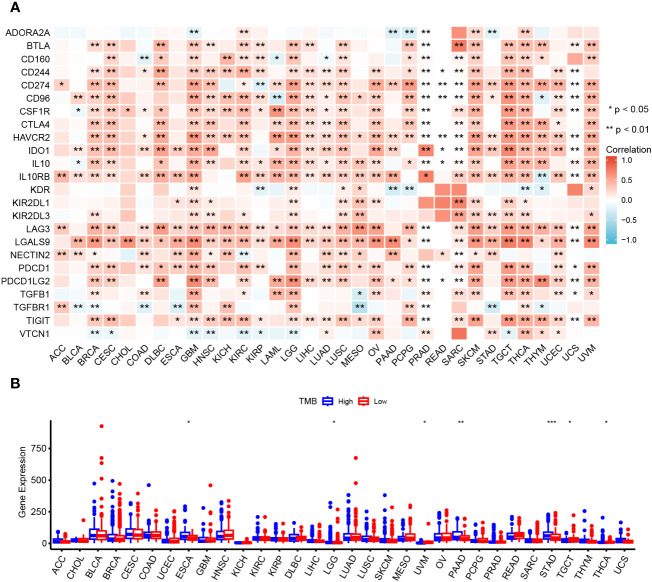
Relationship between OAS1 and immune checkpoint genes and TMB. Correlation of OAS1 expression with immune checkpoint genes expression **(A)** and TMB **(B)** in pan-cancers. *p < 0.05, **p < 0.01, ***p < 0.001.

Our above data suggest that OAS1 may be involved in the immune response in pan-cancer, which indicated that OAS1 might be involved in antitumor immune response. Here, we further explored the correlation between OAS1 expression and the immune checkpoint genes. Our results showed that OAS1 expression in BRCA, GBM, HNSC, KIRC, LGG, LUSC, SARC, SKCM, TGCT, THCA, and UVM were positively associated with various immune checkpoint genes, such as BTLA, CD244, CD247, CD96, CSF1R, CTLA 4, IDO 1, IL 10 and LAG 3, and other immune checkpoint genes (**Figure 8A**). Tumor mutational burden (TMB) is a conspicuous biomarker correlated with the immunotherapy response. The correlation between the OAS1 expression and TMB was examined. High OAS1 patients had higher TMB in ESCA, LGG, PAAD and STAD, whereas patients with high OAS1 expression exhibited lower TMB in UVM, TGCT and THCA (**Figure 8B**). These data indicate that OAS1 may be involved in the tumor immunotherapy response.

### OAS1 regulates the cell cycle, apoptosis, and migration of LUAD and PRAD cells

**Figure 9:**
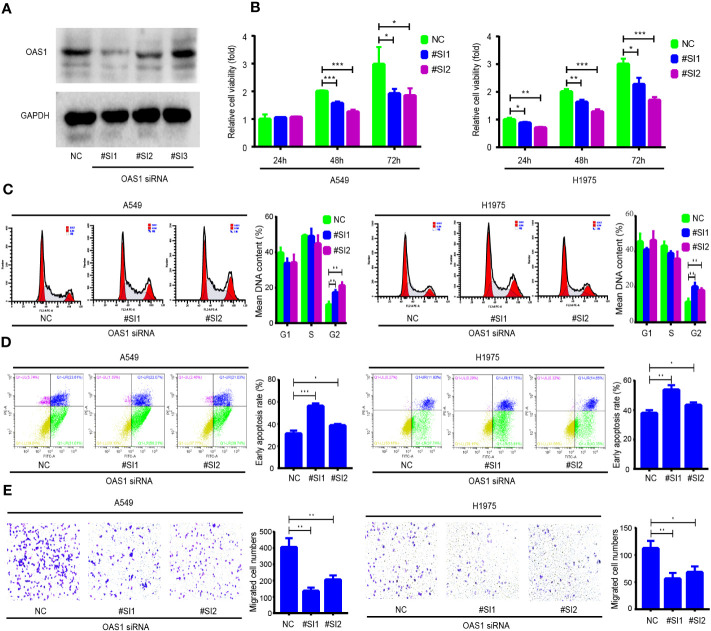
The role of OAS1 on cell proliferation, cell cycle, apoptosis and migration of lung adenocarcinoma (LUAD) cells. **(A)** The silencing efficiency of OAS1 siRNA was examined by Western blot in A549 cells. **(B)** CCK-8 assay was utilized to assess the proliferation rate of A549 cells and H1975 cells, flow cytometry was utilized to examine the cell cycle **(C)** and apoptosis **(D)** of A549 and H1975 cells. **(E)** Transwell was utilized to examine cell migration ability of A549 cells and H1975 cells.

To further examine the role of OAS1 in cancer cells, LUAD (A549 and H1975) and PRAD (PC-3 and C4-2) cell lines were selected for functional experiments. The OAS1-targeting siRNAs were used to silence OAS1 expression, and the inhibition efficiency of OAS1-targeting siRNAs were determined by Western blot. OAS1-targeting siRNAs were significantly reduced OAS1 expression after OAS1 siRNA #SI1 and #SI2 transfection (**Figure 9A** and **Supplementary Figure 1A**). The effects of OAS1 on cell proliferation was examined by CCK-8 assay. We observed that the silence of OAS1 significantly inhibited the proliferative activity of A549 and H1975 cells (**Figure 9B**), as well as PC-3 and C4-2 cells (**Supplementary Figure 1B**). The effects of silence OAS1 expression on the cell cycle and apoptosis in LUAD cells were determined by flow cytometry. The cell cycle of A549, H1975, PC-3 and C4-2 cells arrested in the G2/M phase after OAS1 silencing (**Figure 9C** and **Supplementary Figure 1C**). In addition, silence of OAS1 significantly enhanced cisplatin-induced apoptosis both in LUAD cells and PRAD cells (**Figure 9D** and **Supplementary Figure 1D**), but impaired cell migration (**Figure 9E** and **Supplementary Figure 1E**). These data suggest that OAS1 plays a role in regulating cell cycle, apoptosis and cell migration in cancer cells.

## Discussion

Cancer is one of the most common and dangerous diseases leading to human death. Tumor heterogeneity and drug resistance lead to a poor prognosis and high mortality rate (3). Currently, the most common treatments for tumors are surgery, radiation and adjuvant chemotherapy, but most patients with advanced tumors show a poor prognosis (2). Although immunotherapy occupied an important position in patients with advanced tumors in recent years, the response rate is very low (28). Therefore, the early prevention of cancer and the exploration of effective therapeutic targets are important to improve the prognosis of patients. Previous studies have shown that OAS1 is a multifunctional effector protein, as a member of the OAS protein family, is involved in a range of cell biological processes (7). The expression and activity of OAS1 can be used to evaluate the antiviral effect of interferon in the body (10, 29). Studies have shown that the changes of OAS1 can also be used as a basis to determine the course of some immune diseases (9). It is noteworthy that the latest studies show that OAS1 is associated with COVID-19 incidence (12, 13), so OAS1 may have certain application prospect for clinical evaluations.

In this study, we applied bioinformatics methods to explore the role of OAS1 in pan-cancer. We analyzed the transcription level of OAS1 in pan-cancer based on TIMER, TCGA databases, and also using the CPTAC and HPA databases to explore the protein level of OAS1. In our results, OAS1 expression in BLCA, BRCA, CHOL, ESCA, HNRC, KIRC, KIRC, KIRP, LUAD, READ, THCA, STAD, and UCEC data and showed significantly higher expression in these cancers than in adjacent normal tissues. However, the opposite is true for COAD and KICH. This finding suggests that the level of OAS1 expression varied according to tumor type and plays different role in different tumors.

Further studies revealed that high OAS1 expression was correlated with a poor prognosis in multiple tumor types, including ACC, LGG, LIHC, LUAD, PAAD, PRAD, and UVM. Previous studies showed that OAS1 is correlated with poor prognosis in PAAD, which is consistent with our findings. It is interesting that Zhang Wang’s report (14) shows that OAS1 was associated with the poor prognosis of breast cancer, we did not observe this result in this study. The geographical origin of samples may lead to two different conclusions. The samples of the TCGA database used in this study are mainly from the United States, while the samples of Zhang’s study are from China. We analyzed the differences of OAS1 expression between Caucasians and Asians, data showed that the expression level of OAS1 in Asians was significantly higher than that in Caucasians (data not shown). It may be that only when the expression level of OAS1 is high enough can it affect the prognosis of patients with breast cancer, which may explain why the two results are inconsistent. In addition, high expression of OAS1 in BLCA and SKCM predicts a longer OS. It is worth noting that the expression level of OAS1 in BLCA normal tissue was significantly higher than that in cancer tissue, which may suggest that OAS1 acts as a tumor suppressor gene in BLCA. Study shows that the OAS1 SNP polymorphism is significantly associated with the risk of prostate cancer. The OAS1 rs2660 AA genotype increases the risk of prostate cancer, whereas the GG genotype has a protective effect on prostate cancer (30). Therefore, in addition to expression level changes, the OAS1 SNP polymorphisms may also affect the prognosis of patients in multiple tumors. Unfortunately, this study did not investigate differences in the OAS1 SNP polymorphism in pan-cancers and its impact on patient prognosis. The above researches indicate that OAS1 may be a potential biomarker to predict the prognosis of cancer patients.

Previous studies have confirmed that OAS1 levels alteration affects antiviral activity, cell growth, cell cycle, apoptosis and immune response (7). Furthermore, OAS1 SNPs are associated with the occurrence of several diseases, including diabetes (10), tuberculosis infection (30), Sjogren’s syndrome (31), central nervous system (CNS) involvement of enterovirus 71 infection (32) and multiple sclerosis (33, 34). In current study, we found that OAS1 may be involved in multiple biological processes and signaling pathways, such as biological metabolism, DNA replication, proteasome, cytokine receptor interaction, NK cell-mediated cytotoxic effects, graft versus host disease, processing and presentation of antigen and chemokine receptors binding chemokines, and others using GSEA and KEGG annotation analyses. These data suggest that OAS1 regulates tumorigenesis and progression through multiple mechanisms, including the anti-tumor immune response.

Immune subtypes are closely correlated with the prognosis of tumor patients. A growing number of studies confirmed that C2 and C3 have abundant enrichment of CD8 T cells, while C3, C4, and C6 showed massive enrichment of CD4 T cells. C3 has the best prognosis, which may be associated with high Th17 characteristics in addition to CD8 T cell enrichment (35). C4 and C6 showed macrophage enrichment, high M2 macrophages, and low lymphocyte infiltration, which demonstrated an immunosuppressive TME, and resulting in the worst prognosis. C2 was IFN- γ dominant, with poor survival despite the highest CD8 T and M1 macrophages infiltrates (36). We note that OAS1 expression in 12 cancer types was significantly associated with different immune subtypes. OAS1 was highly expressed in the C6 characteristic population of both tumors (BLCA and KIRP). OAS1 was highly expressed in the C2 characteristic population of nine tumors (BRCA, COAD, HNSC, KIRC, LUAD, READ, STAD, THCA, and UCEC). OAS1 was highly expressed in ESCA patients with C4 characteristics. Interestingly, C6 characteristics were associated with the worst prognosis outcome, however, OAS1 was high expression in C6 in BLCA and with a good prognosis in current study. This is probably because OAS1 may act as a tumor suppressor gene in other biological processes, such as cell proliferation, metastasis and drug resistance in BLCA, although OAS1 protects an immunosuppressive TME in BLCA, and these guesses need to be verified in the future.

In order to investigate the role of OAS1 in tumor immune response, we also analyzed the relationship between OAS1 and immune checkpoint gene expression in pan-cancer. The expression of OAS1 was significantly correlated with the expression of multiple immune checkpoint genes in multiple tumors, including BTLA, CD244, CD247, CD96, CSF1R, CTLA4, IDO1, IL10, PDCD1 and LAG3. OAS1 may affect the anti-tumor immune response by regulating the expression of immune checkpoint genes. Numerous studies confirmed that immune checkpoint genes are important in cancer immunotherapy (37, 38), and a large number of immune checkpoint inhibitors are widely used in clinical tumor therapy or clinical trials (39). An increasing number of studies have demonstrated that TMB is positively correlated with cancer immunotherapy efficacy; the higher the TMB, the better is the efficacy of immune checkpoint inhibitors. The expression of OAS1 was associated with TMB in several tumors in this study. Therefore, OAS1 can be used to predict the therapeutic effect of tumor immunotherapy. These data suggest that OAS1 may play an important role in the response to tumor immunotherapy.

This study also has some drawbacks. Firstly, the survival rates and follow-up times differ among tumor types in the TCGA data. Secondly, the pathologist’s review found that samples with small tumor cell nuclei (less than 60%) in the TCGA data were excluded from the analysis, which raised the possibility that the majority of tumor samples with immune-infiltrating would be removed from the study (36). Thirdly, we did not collect clinical samples to verify the expression level of OAS1 in each type of tumors and the relationship between patient prognosis and OAS1 expression. Finally, we only performed a functional enrichment analysis, and we did not use the *in vitro* and *in vivo* experiments to verify the functions of OAS1 in each tumor type.

In summary, OAS1 is differentially expressed in pan-cancers and is correlated with patient prognosis. OAS1 can be used as a tumor prognostic marker. OAS1 correlated with molecular subtypes or immune subtypes in numerous tumors. The OAS1 expression was significantly associated with the expression of multiple immune checkpoint genes and TMB, and these data indicate that OAS1 may be involved in regulating the antitumor immune response. This study added a new dimension to our comprehensive understanding of OAS1 in tumor initiation and progression, and provided bioinformatics evidence and experimental evidence for application of OAS1 as a target gene in clinical tumor therapy in the future.

## Data availability statement

Publicly available datasets were analyzed in this study. This data can be found here: https://cancergenome.nih.gov.

## Ethics statement

Ethical approval was not required for the studies on humans in accordance with the local legislation and institutional requirements because only commercially available established cell lines were used.

## Author contributions

Conception and design of the work: ZL; Experiments, analysis and interpretation of data: SJ and XD; Data collection: ML; Drafting the article: LZ and JC; Literature search and draft revision: CT and YY. All authors contributed to the article and approved the submitted version.
