# Supplementary material for: Pan-cancer analysis identified OAS1 as a potential prognostic biomarker for multiple tumor types
Source: Front Oncol. 2023 Sep 6;13:1207081. doi: 10.3389/fonc.2023.1207081 (PMC10511872; doi:10.3389/fonc.2023.1207081)
Supplement: Supplementary file 1 [file DataSheet_1.docx]

**Supplementary Figures**

Supplementary Figure 1


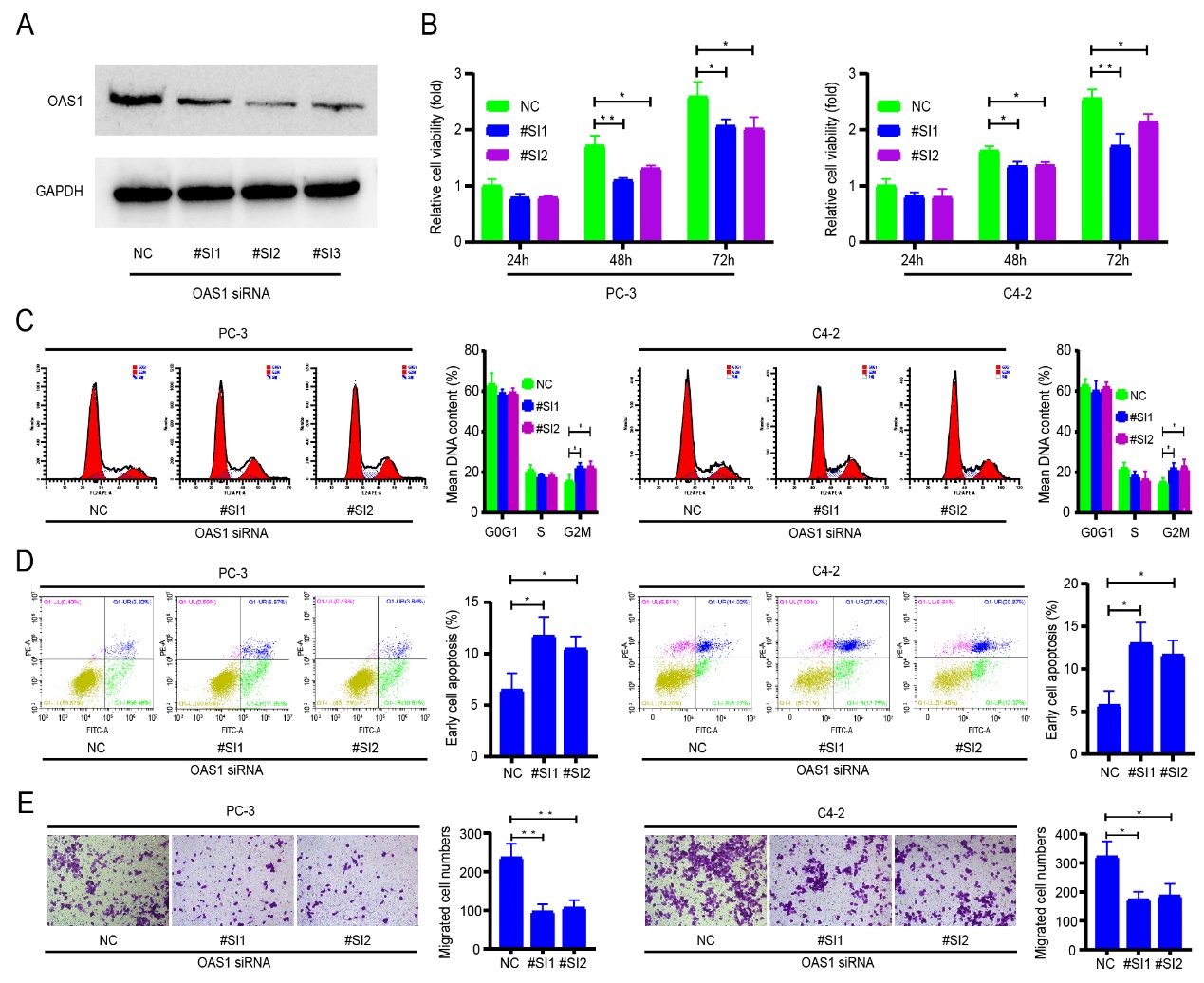


Supplementary Figure 1 **|** The role of OAS1 on cell proliferation, cell cycle，apoptosis and migration of Prostate adenocarcinoma (PRAD) cells. (A) The silencing efficiency of OAS1 siRNA was examined by Western blot in PC-3 cells. (B) CCK-8 assay was utilized to assess the proliferation rate of PC-3 and C4-2 cells, ﬂow cytometry was utilized to examine the cell cycle (C) and apoptosis (D) of PC-3 and C4-2 cells. (E) Transwell was utilized to examine cell migration ability of PC-3 and C4-2 cells.
